# Supplementary material for: Multiple Cofactor Engineering Strategies to Enhance Pyridoxine Production in Escherichia coli
Source: Microorganisms. 2024 May 3;12(5):933. doi: 10.3390/microorganisms12050933 (PMC11123869; doi:10.3390/microorganisms12050933)
Supplement: Supplementary file 1 [file microorganisms-12-00933-s001.zip › Supporting Information.pdf]

Supporting information for:

Multiple cofactor engineering strategies to enhance pyridoxine production in *Escherichia coli*

Lijuan Wu [1,2,3,4,†], Jinlong Li [2,3,4,5,†], Yahui Zhang [1,2,3,4], Zhizhong Tian [2,3,4], Zhaoxia Jin [1,*], Linxia Liu [2,3,4,*] and Dawei Zhang [2,3,4,5]

1 School of Biological Engineering, Dalian Polytechnic University, Dalian, China
2 Tianjin Institute of Industrial Biotechnology, Chinese Academy of Sciences, Tianjin, China
3 National Center of Technology Innovation for Synthetic Biology, Tianjin, China
4 Key Laboratory of Engineering Biology for Low-Carbon Manufacturing, Tianjin Institute of Industrial Bio-technology, Chinese Academy of Sciences, Tianjin, China
5 University of Chinese Academy of Sciences, Beijing, China
* Correspondence: jinzx2018@163.com (Z.J.); liulx@tib.cas.cn (L.L.)
† These authors contributed equally to this work.

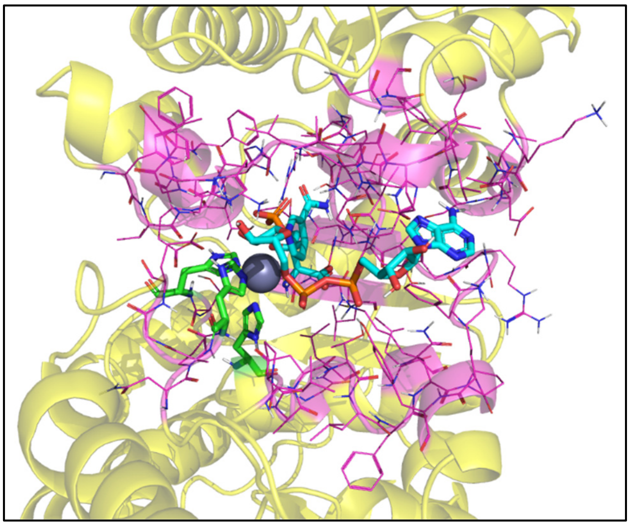

Figure S1. Seventy-one residues (highlighted in magenta lines) within a 6 Å radius around TSA (shown as cyan sticks), excluding the three histidine residues (represented by green sticks) responsible for ion stabilization (depicted as gray balls), were chosen for design. The 71 residues are as follows: G16, I17, G18, L21, T118, G119, P120, V121, H122, K123, G124, V125, F133, T134, G135, H136, T137, E138, F139, F140, M151, M152, L153, T165, N209, P210, A212, G213, E214, L244, P245, A246, D247, T248, L249, F250, Q251, P252, K253, Y254, G269, L270, L273, K274, F278, G279, R280, G281, V282, N283, I284, T285, R292, T293, S294, V295, D296, H297, G298, T299, A300, L301, E302, L303, K308, A309, D310, V311, G312, S313, F314.

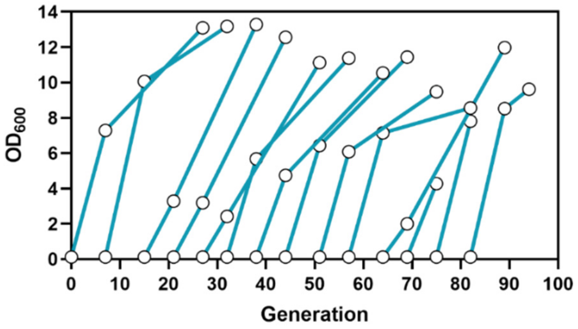

Figure S2. The cell growth (OD_{600}) through sequentially subculturing from 1st to 100th generations.

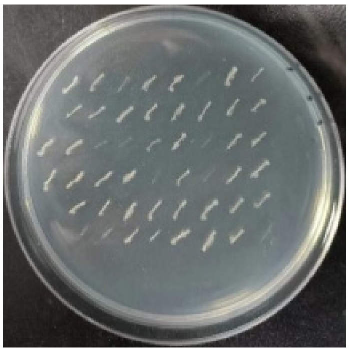

Figure S3. Plasmid stability test. The fermentation sample was placed on a non-selective plate, incubated at 37°C for 16-20 h, and a single colony was randomly selected and spotted on the LB plate supplemented with kanamycin, chloramphenicol and ampicillin. The colonies are counted and the percentage stability was calculated by determining the ratio of the number of colonies on the growth plate to the total number of spots.

Table S1. The binding energy of wild-type pdxA and F140I in different states with 4HTP and NAD^+.

Binding energy (MMGBSA)<br>unit: kcat/mol	4HTP	NAD^+	Total
WT	-96.71	-45.87	-142.58
WT_precatalytic	-132.34	-51.51	-183.84
F140I	-101.82	-37.42	-139.24
F140I_precatalytic	-164.18	-44.67	-208.85

Table S2. Primers used in this study.

names	Sequences (5′-3′)
xfp-up-F	GACGAAGAATCCATGGGCCTGTCGTTGGTGATATGCGCAAGC
xfp-up-R	GCTAGCATTATACCTAGGACTGAGCTAGCTGTCAAAACTGAACGG TTAAACATGCCAC
xfp-down-F	CCTTCGGGTGGGCCTTTCTGCGTCTTGCCGCTCCCCTGC
xfp-down-R	GGTGAGAATCCAAGCTTCCATTCAGTTACACATGCTGCCGGAAAT C
xfp-v1-F	TGAATGGAAGCTTGGATTCTCACC
xfp-N20-R	CTGGATCATAGGCTGGAACAGCTAAGATCTGACTCCATAACAGAG TACTC
xfp-v2-R	ACAGGCCCATGGATTCTTCGTC
xfp-N20-F	TGTTCCAGCCTATGATCCAGGTTTTAGAGCTAGAAATAGCAAGTTA AAATAAGGCTAG
Cas9-test-F	ATGGCACATAGCCTTGCTCAAAT
Cas9-test-R	GGATTTGTTCAGAACGCTCGGTT
xfp-test-F	GTTATCGCGCAAGACGCGAG
xfp-test-R	GGCGCAATTCATTGATGCAGC
F140I-F	CACCGAATTCATCGAAGAACGTTCTCAGGCTAAAAAAG
F140I-R	CTTCGATGAATTCGGTGTGACCGGTGA
T165C-F	TTGCTCTGGCTACCTGCCACCTGCCGCTGCGTGAC
T165C-R	GCAGGTAGCCAGAGCAACACGCA
PdxA-test-F	TTGTACACGGCCGCATAATC
PdxA-test-R	GATTATGCGGCCGTGTACAA
Pro-spNox-up-F	ACAGACGAAGAATCCATGGGCCTGTAACACTGTCTGTTGTTCACTT TTTCAGG
Pro-spNox-up-R	GGCATCCCCGGGGTGTCAAATTTGGTATAAGTTGATGGGAATGATG TCTGCTTCAGAGTATTGCAGATGCC
Pro-spNox-down-F	GCGAAAGACTAAATGATCAGGCAGAAGATTCTACAGC
Pro-spNox-down-R	TTTATTGGTGAGAATCCAAGCTTCCATTCACAATAATCACGGTGGC GGTATTCAC
Pro-spNox-F	CTTATACCAAATTTGACACCCCGGGGATGCCATAAACCTATCCCCC ACCCGTTTTTTGGGCTAACAGGAGG
Pro-spNox-R	GAGCTGCTGTAGAATCTTCTGCCTGATCATTTAGTCTTTCGCACCCA GTGCTGC
Pro-spNox-v1-F	CGTGATTATTGTGAATGGAAGCTTGGATTCTCACC
Pro-spNox-N20-R	ACTGGGCCCTAAGTGTATCAAAGCTAAGATCTGACTCCATAACAG AGTACTC
Pro-spNox-v2-R	AGACAGTGTTACAGGCCCATGGATTCTTCGTC

Pro-spNox-N20-F	GCTTTGATACACTTAGGGCCCAGTTTTAGAGCTAGAAATAGCAAGTTAAAATAAGGCTAGTC
spNox-up-F	CAGACGAAGAATCCATGGGCCTGTAACACTGTCTGTTGTTCACTTTTTCAGGCG
spNox-up-R	CAAAAAACGGGTGCTAGCACAATACCTAGGACTGAGCTAGCCGTCAAGTCTGCTTCAGAGTATTGCAGATG
spNox-down-F	ACCATGGCAGCACTGGGTGCGAAAGACTAAATGATCAGGCAGAAGATTCTACAGCAG
spNox-down-R	TTTATTGGTGAGAATCCAAGCTTCCATTCACAATAATCACGGTGGCGGTATTCAC
spNox-F	TCCTAGGTATTGTGCTAGCACCCGTTTTTTGGGCTAACAGGAGGAATTAACCATGTCTAAAATCGTTGTTG
spNox-R	AGGAGCTGCTGTAGAATCTTCTGCCTGATCATTTAGTCTTTCGCACCCAGTGC
spNox-v1-F	TGAATGGAAGCTTGGATTCTCACC
spNox-N20-R	GCGCGTGACTACATTGAGTCAAGTTTTAGAGCTAGAAATAGCAAGTTAAAATAAGGCTAGTC
spNox-v2-R	ACAGGCCCATGGATTCTTCGTC
spNox-N20-F	ACTTGACTCAATGTAGTCACGCGCTAAGATCTGACTCCATAACAGAGTACTC
spNox-test-F	CGGCGCGGGAAATTCTTAAA
spNox-test-R	TGGCTAAATGATGACGTCGTAG
GapC-up-F	GACGAAGAATCCATGGGCCTGTATGGTGCTGCCGGTCGCGAT
GapC-up-R	CAATCCTGTGCCTAAGCATTACGCGACTGAATTTACTGCGTACTTCGACAACC
GapC-down-F	CTGGCTTACTTCGCTAAAATCGCTAAATAATTAGATTTGACTGAAATCGTACAGTAAAAAGCG
GapC-down-R	GGTGAGAATCCAAGCTTCCATTCACATGCGTGTCCCAGGTGTC
GapC-F	CATTAATGGTTGTCGAAGTACGCAGTAAATTCAGTCGCGTAATGCTTAGGCAC
GapC-R	ACGATTTCAGTCAAATCTAATTATTTAGCGATTTTAGCGAAGTAAGCCAGGGTAC
GapC-v1-F	GACACGCATGTGAATGGAAGCTTGGATTCTCACCAATAAAAAAC
GapC-N20-R	GCTCTAAAACGGAAGGACTCGTCACCCTCGGCTAAGATCTGACTCCATAACAGAGTACTCG
GapC-v2-R	CCGGCAGCACCATACAGGCCCATGGATTCTTCGTCTGTTT
GapC-N20-F	CGAGGGTGACGAGTCCTTCCGTTTTAGAGCTAGAAATAGCAAGTTAAAATAAGGCTAGTC
GapN-up-F	GACGAAGAATCCATGGGCCTGTATGGTGCTGCCGGTCGCGAT
GapN-up-R	CAATCCTGTGCCTAAGCATTACGCGACTGAATTTACTGCGTACTTCGACAACC
GapN-F	CATTAATGGTTGTCGAAGTACGCAGTAAATTCAGTCGCGTAATGCTTAGGCAC

GapN-R	ACGATTTCAGTCAAATCTAATTATTTGATGTCGAAAACAACAGATTTAACGGTG
GapN-down-F	TGTTGTTTTCGACATCAAATAATTAGATTTGACTGAAATCGTACAGTAAAAAGCG
GapN-down-R	GGTGAGAATCCAAGCTTCCATTCACATGCGTGTCCCAGGTGTC
GapN-v1-F	GACACGCATGTGAATGGAAGCTTGGATTCTCACCAATAAAAAAC
GapN-N20-R	GCTCTAAAACGGAAGGACTCGTCACCCTCGGCTAAGATCTGACTCCATAACAGAGTACTCG
GapN-v2-R	CCGGCAGCACCATACAGGCCCATGGATTCTTCGTCTGTTT
GapN-N20-F	CGAGGGTGACGAGTCCTTCCGTTTTAGAGCTAGAAATAGCAAGTTAAAATAAGGCTAGTC
GapN/C-test-F	ATTGAGGCCGTCTGTCTTGG
GapN/C-test-R	CGCCGGAAGCGTTCATAAAG

Table S3. Sequences of heterologous genes.

names	Sequences (5′-3′)
xfp	ATGGCCATGACTTCACCAGTAATAGGAACACCCTGGAAGAAATTGAATGCG<br>CCAGTGAGCGAAGAGGCTCTGGAGGGCGTAGATAAGTATTGGCGCGTGGC<br>CAACTATCTGAGCATCGGCCAGATTTATCTGCGTTCCAACCCGCTCATGAAA<br>GAACCATTCACCCGTGAGGACGTGAAGCATCGCCTGGTTGGTCACTGGGGA<br>ACGACCCCGGGTCTGAACTTTCTGATTGGCCACATTAACAGATTCATCGCGG<br>ACCACGGTCAGAATACCGTTATCATTATGGGTCCAGGTCACGGCGGTCCGG<br>CTGGCACCAGCCAGAGCTACCTGGACGGAACCTATACCGAAACCTTTCCGA<br>AGATCACCAAAGATGAGGCAGGCCTGCAAAAATTTTTCCGCCAGTTCTCAT<br>ACCCGGGGGGCATCCCGTCTCATTTCGCACCGGAAACCCCGGGTAGCATTC<br>ACGAAGGTGGCGAATTGGGCTACGCATTGTCCCACGCCTACGGCGCGATCA<br>TGGATAATCCAAGCTTGTTTGTTCCGGCTATCGTGGGCGATGGCGAGGCGG<br>AGACTGGTCCGCTGGCGACCGGTTGGCAGTCTAATAAGTTGGTCAATCCGC<br>GTACCGACGGTATCGTTCTTCCGATCCTTCACCTGAACGGCTATAAAATTGC<br>GAACCCGACCATCCTGAGCCGTATCAGCGATGAGGAACTGCATGAATTCTT<br>TCATGGTATGGGTTACGAGCCGTATGAGTTCGTTGCTGGTTTTGACGATGAG<br>GACCACATGTCCATTCACCGTCGTTTTGCCGAATTATGGGAAACGATTTGGG<br>ATGAGATCTGTGATATCAAAGCAACCGCGCAGACCGATAACGTTCACCGTC<br>CGTTTTACCCGATGCTGATTTTTCGCACGCCAAAAGGCTGGACTTGCCCGAA<br>GTACATCGACGGCAAAAAGACCGAAGGCAGCTGGCGTTCTCACCAAGTTC<br>CGCTCGCGTCTGCCCGTGATACTGAAGCGCACTTCGAAGTGCTGAAGAACT<br>GGCTGGAGAGCTACAAGCCTGAAGAACTGTTCGATGCAAACGGCGCAGTT<br>AAAGACGACGTTCTGGCGTTTATGCCGAAGGGCGAATTGCGTATTGGTGCG<br>AACCCTAACGCAAATGGTGGTGTCATTCGCAACGACCTGAAGTTACCGAAC<br>CTTGAGGACTACGAGGTAAAGGAAGTGGCTGAGTATGGTCACGGCTGGGG<br>TCAACTGGAAGCAACTCGTACCCTGGGTGCGTACACCCGTGACATCATTAA<br>GAACAACCCGCGTGATTTTCGCATCTTCGGTCCGGATGAAACCGCGTCGAA<br>CCGCCTGCAGGCATCCTACGAGGTTACGAACAAACAGTGGGACGCGGGTT<br>ACATTAGCGATGAGGTGGATGAGCACATGCATGTTAGCGGACAGGTTGTTG<br>AACAGCTGTCGGAACACCAAATGGAAGGTTTTTTGGAAGCGTACCTGCTGA<br>CGGGTCGTCATGGCATCTGGAGCAGCTATGAGTCCTTTGTTCACGTCATTGA<br>CAGCATGCTGAATCAGCATGCAAAATGGCTGGAAGCCACGGTGCGTGAAA<br>TTCCGTGGCGTAAACCGATCGCGAGCATGAATCTGCTCGTGTCGAGCCACG<br>TGTGGCGCCAAGATCATAACGGCTTCAGCCATCAAGATCCGGGCGTGACTT<br>CAGTTCTGCTGAACAAATGCTTTCACAACGATCACGTGATCGGTATCTACTT<br>CGCTACGGACGCGAACATGCTCTTGGCCATCGCTGAGAAGTGCTATAAAAG<br>CACCAATAAGATCAACGCTATTATTGCGGGCAAGCAACCGGCGGCGACCTG<br>GTTAACCCTGGATGAGGCGCGTGCAGAATTGGAGAAGGGCGCGGCTGCTT<br>GGGATTGGGCATCCACCGCGAAAAACAATGACGAAGCGGAGGTCGTGCTG<br>GCAGCTGCGGGTGATGTCCCGACCCAAGAAATTATGGCAGCTTCCGACAAA<br>CTGAAAGAGCTGGGTGTGAAATTCAAAGTGGTGAACGTGGCCGATCTGCTG<br>TCGCTGCAATCTGCAAAAGAGAACGACGAGGCGCTGACCGACGAGGAATT

	CGCTGACATCTTCACCGCGGACAAACCGGTCTTATTCGCCTACCATAGCTAC<br>GCCCACGACGTTCGTGGTCTTATCTATGACCGCCCTAATCATGACAATTTCA<br>ACGTGCATGGTTATGAAGAGGAAGGTTCTACCACCACCCCGTACGATATGG<br>TTCGTGTTAATCGCATAGACCGTTACGAGTTGACGGCGGAGGCTCTGCGCAT<br>GATTGATGCAGACAAATACGCGGACAAGATCGATGAGTTGGAGAAGTTCC<br>GCGACGAGGCGTTTCAGTTCGCTGTCGATAATGGTTACGACCATCCGGATTA<br>TACAGACTGGGTTTATTCCGGTGTTAATACCGATAAGAAGGGTGCTGTGACC<br>GCAACGGCGGCTACTGCGGGTGACAATGAATAA
Sp*Nox*	TCTAAAATCGTTGTTGTAGGTGCAAACCACGCGGGTACTGCATGCATCAAG<br>ACCATGCTGACCAATTACGGTGATGCAAACGAAATTGTGGTATTCGACCAG<br>AACAGCAACATCTCTTTTCTGGGCTGTGGTATGGCGCTGTGGATCGGTGAAC<br>AGATTGCGGGTCCGGAAGGCCTGTTCTATAGCGACAAAGAAGAACTGGAA<br>TCCCTGGGCGCTAAAGTTTACATGGAATCCCCGGTGCAATCTATCGACTATG<br>ACGCAAAAACTGTTACCGCCCTGGTAGATGGCAAAAACCACGTAGAGACC<br>TACGATAAACTGATCTTTGCGACTGGTTCTCAGCCTATCCTGCCGCCGATTA<br>AAGGCGCAGAAATCAAGGAGGGTTCTCTGGAATTCGAAGCCACTCTGGAA<br>AATCTGCAGTTCGTTAAACTGTACCAGAACTCTGCTGACGTTATCGCGAAA<br>CTGGAAAATAAAGACATTAAACGTGTCGCTGTGGTTGGTGCGGGCTATATC<br>GGCGTTGAACTGGCAGAAGCCTTCCAGCGCAAAGGCAAAGAAGTTGTTCT<br>GATTGACGTGGTTGACACCTGCCTGGCTGGTTACTACGATCGTGACCTGACG<br>GACCTGATGGCTAAAAACATGGAGGAACACGGTATTCAGCTGGCCTTTGGT<br>GAAACCGTTAAAGAAGTTGCGGGCAACGGTAAAGTTGAGAAAATCATTAC<br>TGACAAAAACGAATACGATGTAGACATGGTAATCCTGGCTGTGGGTTTTCGT<br>CCGAATACGACCCTGGGTAATGGTAAAATTGACCTGTTCCGCAACGGCGCG<br>TTTCTGGTTAACAAACGTCAAGAAACCTCTATTCCGGGTGTATACGCTATTG<br>GCGATTGCGCAACGATCTATGACAACGCAACTCGTGATACCAACTACATCG<br>CACTGGCCTCTAACGCGGTTCGCACTGGCATCGTTGCGGCACACAACGCTT<br>GCGGCACCGATCTGGAAGGTATCGGCGTGCAGGGCTCTAACGGCATCTCCA<br>TTTATGGCCTGCACATGGTTTCTACCGGCCTGACCCTGGAAAAGGCTAAAC<br>GTCTGGGTTTCGATGCTGCCGTTACCGAGTATACTGATAACCAGAAGCCAG<br>AATTCATCGAACACGGCAACTTCCCTGTGACGATCAAGATCGTTTACGATA<br>AGGATTCCCGTCGTATTCTGGGCGCGCAGATGGCAGCACGTGAAGACATGT<br>CTATGGGTATTCATATGTTCTCTCTGGCAATTCAGGAAGGTGTTACGATTGAG<br>AAGCTGGCTCTGACCGACATCTTCTTCCTGCCGCACTTCAACAAACCGTAC<br>AACTATATCACCATGGCAGCACTGGGTGCGAAAGACTAA
gapN	ATGACCAAACAGTACAAAAACTACGTTAACGGTGAATGGAAACTGTCTGA<br>AAACGAAATCAAAATCTACGAACCGGCTTCTGGTGCTGAACTGGGTTCTGT<br>TCCGGCTATGTCTACCGAAGAAGTTGACTACGTTTACGCTTCTGCTAAAAAA<br>GCTCAGCCGGCTTGGCGTTCTCTGTCTTACATCGAACGTGCTGCTTACCTGC<br>ACAAAGTTGCTGACATCCTGATGCGTGACAAAGAAAAAATCGGTGCTGTTC<br>TGTCTAAAGAAGTTGCTAAAGGTTACAAATCTGCTGTTTCTGAAGTTGTTCG<br>TACCGCTGAAATCATCAACTACGCTGCTGAAGAAGGTCTGCGTATGGAAGG<br>TGAAGTTCTGGAAGGTGGTTCTTTCGAAGCTGCTTCTAAAAAAAAAATCGC<br>TGTTGTTCGTCGTGAACCGGTTGGTCTGGTTCTGGCTATCTCTCCGTTCAACT

	ACCCGGTTAACCTGGCTGGTTCTAAAATCGCTCCGGCTCTGATCGCTGGTAA<br>CGTTATCGCTTTCAAACCGCCGACCCAGGGTTCTATCTCTGGTCTGCTGCTG<br>GCTGAAGCTTTCGCTGAAGCTGGTCTGCCGGCTGGTGTTTTCAACACCATCA<br>CCGGTCGTGGTTCTGAAATCGGTGACTACATCGTTGAACACCAGGCTGTTA<br>ACTTCATCAACTTCACCGGTTCTACCGGTATCGGTGAACGTATCGGTAAAAT<br>GGCTGGTATGCGTCCGATCATGCTGGAACTGGGTGGTAAAGACTCTGCTATC<br>GTTCTGGAAGACGCTGACCTGGAACTGACCGCTAAAAACATCATAGCGGGT<br>GCGTTCGGTTACTCTGGCCAGCGTTGCACCGCTGTTAAACGTGTTCTGGTTA<br>TGGAATCTGTTGCTGACGAACTGGTTGAAAAAATCCGTGAAAAAGTTCTGG<br>CTCTGACCATCGGTAACCCGGAAGACGACGCTGACATCACCCCGCTGATCG<br>ACACCAAATCTGCTGACTACGTTGAAGGTCTGATCAACGACGCTAACGACA<br>AAGGTGCTGCTGCTCTGACCGAAATCAAACGTGAAGGTAACCTGATCTGCC<br>CGATCCTGTTCGACAAAGTTACCACCGACATGCGTCTGGCTTGGGAAGAAC<br>CGTTCGGTCCGGTTCTGCCGATCATCCGTGTTACCTCTGTTGAAGAAGCTAT<br>CGAAATCTCTAACAAATCTGAATACGGTCTGCAGGCTTCTATCTTCACCAAC<br>GACTTCCCGCGTGCTTTCGGTATCGCTGAACAGCTGGAAGTTGGTACCGTTC<br>ACATCAACAACAAAACCCAGCGTGGTACCGACAACTTCCCGTTCCTGGGTG<br>CTAAAAAATCTGGTGCTGGTATCCAGGGTGTTAAATACTCTATCGAAGCTAT<br>GACCACCGTTAAATCTGTTGTTTTCGACATCAAATAA
gapC	ATGGCTAAAATCGCTATCAACGGTTTCGGTCGTATCGGTCGTCTGGCTCTGC<br>GTCGTATCCTGGAAGTTCCGGGTCTGGAAGTTGTTGCTATCAACGACCTGAC<br>CGACGCTAAAATGCTGGCTCACCTGTTCAAATACGACTCTTCTCAGGGCAG<br>GTTCAACGGTGAAATAGAAGTTAAAGAAGGTGCTTTCGTTGTTAACGGTAA<br>AGAAGTTAAAGTTTTCGCTGAAGCTGACCCGGAAAAACTGCCGTGGGGTG<br>ACCTGGGTATCGACGTTGTTCTGGAATGCACCGGTTTCTTCACCAAAAAAG<br>AAAAAGCTGAAGCTCACGTTCGTGCTGGTGCTAAAAAAGTTGTTATCTCTG<br>CTCCGGCTGGTAACGACCTGAAAACCATCGTTTTCAACGTTAACAACGAAG<br>ACCTGGACGGTACCGAAACCGTTATCTCTGGTGCTTCTTGCACCACCAACT<br>GCCTGGCTCCGATGGCTAAAGTTCTGAACGACAAATTCGGTATCGAAAAAG<br>GTTTCATGACCACCATCCACGCTTTCACCAACGACCAGAACACCCTGGACG<br>GTCCGCACCGTAAAGGTGACCTGCGTCGTGCTCGTGCTGCTGCTGTTTCTAT<br>CATCCCGAACTCTACCGGTGCTGCTAAAGCTATCTCTCAGGTTATCCCGGAC<br>CTGGCTGGTAAACTGGACGGTAACGCTCAGCGTGTTCCGGTTCCGACCGGT<br>AGTATCACCGAGCTGGTAAGCGTTCTGAAAAAAAAAGTTACCGTTGAAGA<br>AATCAACGCTGCTATGAAAGAAGCTGCTGACGAATCTTTCGGTTACACCGA<br>AGACCCGATCGTTTCTGCTGACGTTGTTGGTATCAACTACGGTTCTCTGTTCG<br>ACGCTACCCTGACCAAAATCGTTGACGTTAACGGTTCTCAGCTGGTTAAAA<br>CCGCTGCTTGGTACGACAACGAAATGTCTTACACCTCTCAGCTGGTTCGTAC<br>CCTGGCTTACTTCGCTAAAATCGCTAAATAA

Table S4. The PN titer of the mutants used in this study.

Strain	Description	Titers(mg/L)
LL006	MG1655, Δ*pdxH*::*pdxST*-2 (Bsu), Δ*pta*::Ptac-*pdxP* (Eme)	11.6±2.1
WL01	LL006, Δ*ldhA*::*xfp* (Blo)	14.6±0.1
WL02	LL006 harboring p15ASI-Ptac-*epd* (Gni)-*pdxB* (Eco)-*dxs* (Eme)-$P_{J231119}$-*serC* (Eco), pRSFDuet-1_P3-*pdxA2*-*pdxJ1*	267.0±5.0
WL03	WL01 harboring p15ASI-Ptac-*epd* (Gni)-*pdxB* (Eco)-*dxs* (Eme)-$P_{J231119}$-*serC* (Eco), pRSFDuet-1_P3-*pdxA2*-*pdxJ1*	332.0±1.1
WL25 (G119C)	WL01 harboring p15ASI-Ptac-*epd* (Gni)-*pdxB* (Eco)-*dxs* (Eme)-$P_{J231119}$-*serC* (Eco), pRSFDuet-1_P3-*pdxA4* (G119C)-*pdxJ1*	224.8±7.0
WL12 (F314L)	WL01 harboring p15ASI-Ptac-*epd* (Gni)-*pdxB* (Eco)-*dxs* (Eme)-$P_{J231119}$-*serC* (Eco), pRSFDuet-1_P3-*pdxA5* (F314L)-*pdxJ1*	274.5±17.2
WL10 (F140I)	WL01 harboring p15ASI-Ptac-*epd* (Gni)-*pdxB* (Eco)-*dxs* (Eme)-$P_{J231119}$-*serC* (Eco), pRSFDuet-1_P3-*pdxA3* (F140I)-*pdxJ1*	367.4±66.1
WL78 (I284V/L249M)	WL01 harboring p15ASI-Ptac-*epd* (Gni)-*pdxB* (Eco)-*dxs* (Eme)-$P_{J231119}$-*serC* (Eco), pRSFDuet-1_P3-*pdxA6* (I284V/L249M)-*pdxJ1*	261.9±12.4
WL74 (L303M/G213C)	WL01 harboring p15ASI-Ptac-*epd* (Gni)-*pdxB* (Eco)-*dxs* (Eme)-$P_{J231119}$-*serC* (Eco), pRSFDuet-1_P3-*pdxA7* (L303M/G213C)-*pdxJ1*	225.2±43.9
WL77 (A309C/P252K)	WL01 harboring p15ASI-Ptac-*epd* (Gni)-*pdxB* (Eco)-*dxs* (Eme)-$P_{J231119}$-*serC* (Eco), pRSFDuet-1_P3-*pdxA8* (A309C/P252K)-*pdxJ1*	220.6±11.5
WL82 (H122L/G213C)	WL01 harboring p15ASI-Ptac-*epd* (Gni)-*pdxB* (Eco)-*dxs* (Eme)-$P_{J231119}$-*serC* (Eco), pRSFDuet-1_P3-*pdxA9* (H122L/G213C)-*pdxJ1*	324.4±29.1
WL22 (H136N)	WL01 harboring p15ASI-Ptac-*epd* (Gni)-*pdxB* (Eco)-*dxs* (Eme)-$P_{J231119}$-*serC* (Eco), pRSFDuet-1_P3-*pdxA2* (H136N)-*pdxJ1*	291.39±16.6
WT	WL01 harboring p15ASI-Ptac-*epd* (Gni)-*pdxB* (Eco)-*dxs* (Eme)-$P_{J231119}$-*serC* (Eco), pRSFDuet-1_P3-*pdxA*-*pdxJ1*	234.05±2.7
WL04-0mM Ara	WL01 harboring p15ASI-Ptac-*epd* (Gni)-*pdxB* (Eco)-*dxs* (Eme)-$P_{J231119}$-*serC* (Eco), pRSFDuet-1_P3-*pdxA3* (F140I)-*pdxJ1*, pBAD-SpNox	462.1±5.1
WL04-0.5mM Ara		437.9±9.7
WL04-1mM Ara		413.6±14.4
WL03-5g/L Glu	WL01 harboring p15ASI-Ptac-*epd* (Gni)-*pdxB* (Eco)-*dxs* (Eme)-$P_{J231119}$-*serC* (Eco), pRSFDuet-1_P3-*pdxA2*-*pdxJ1*	437.9±2.0
WL04-5g/L Glu	WL01 harboring p15ASI-Ptac-*epd* (Gni)-*pdxB* (Eco)-*dxs* (Eme)-$P_{J231119}$-*serC* (Eco), pRSFDuet-1_P3-*pdxA3* (F140I)-*pdxJ1*, pBAD-SpNox	479.4±1.6
WL08	WL06 harboring p15ASI-Ptac-*epd* (Gni)-*pdxB* (Eco)-*dxs* (Eme)-$P_{J231119}$-*serC* (Eco), pRSFDuet-1_P3-*pdxA3* (F140I)-*pdxJ1*	139.0±3.3
WL09	WL07 harboring p15ASI-Ptac-*epd* (Gni)-*pdxB* (Eco)-*dxs*	582.4±70.3

	(Eme)-$P_{J231119}$-*serC* (Eco), pRSFDuet-1_P3-*pdxA3* (F140I)-*pdxJ1*	
WL158	WL156 harboring p15ASI-Ptac-*epd* (Gni)-*pdxB* (Eco)-*dxs* (Eme)-$P_{J231119}$-*serC* (Eco), pRSFDuet-1_P3-*pdxA3* (F140I)-*pdxJ1*	664.3±5.0
WL159	WL157 harboring p15ASI-Ptac-*epd* (Gni)-*pdxB* (Eco)-*dxs* (Eme)-$P_{J231119}$-*serC* (Eco), pRSFDuet-1_P3-*pdxA3* (F140I)-*pdxJ1*	676.6±32.7

Supplementary Method:

Molecular Dynamics Simulation

The complex structure of PdxA and the mutant complex bound to HTP and NAD^+ was simulated with the PDB entries 1PS6 and 6XMY as reference structures[1]. All atom molecular dynamics simulations have been performed using AMBER20 molecular dynamics package [2]. The bonded and non-bonded description of the interactions between the various atoms have been generated using the AMBER20 force fields, which include the ff14SB force field parameters. The ANTECHAMBER module and GAFF2 with AM1-BCC charges [3] are used to obtain force field parameters for ligands. Initially, we performed a series of energy minimization steps to eliminate any bad contacts in the initially built structures. During the minimization, protein (@CA,O,N,C) were restrained with harmonic force constants of 20 kcal/mol. The minimization process involves 5000 steps of steepest descent followed by 5000 steps of conjugate gradient method. After the energy minimization, the system was slowly heated up to 310 K in 100 ps MD using 1 fs integration time step, while restraining the solute with 20 kcal/mol harmonic force constant. After this, we performed 15 ps NPT equilibration of the structures with no harmonic restraints. And then, 20 ns constrained MD simulations were executed, so that the ligands were in a reasonable position to react. Finally, 100 ns NPT production simulations with ion constrained were performed at 310 K and 1 atm pressure with 2 fs integration time step. We have implemented periodic boundary condition across the system using a TIP3P water box [4]. We used the Particle Mesh Ewald (PME) techniques integrated with the AMBER package to account for the long range component of the electrostatic interactions [5]. During the dynamics, all the bonds involving hydrogen are restrained using

the SHAKE algorithm [6]. Langevin thermostat with collision frequency of 1/ps is used to maintain the constant temperature while the pressure was controlled by anisotropic Monte-Carlo barostat [7]. The accelerated GPU version of PMEMD [8] was performed on NVIDIA GeForce 20 Series cards. We have employed CPPTRAJ [9] functionality of AMBERTOOLs [6] to perform various analyses on the equilibrium MD simulation trajectories. The images and graphics of the structures shown here were generated using the software package PyMOL [10].

Computational Enzyme Redesign

The transition state analog was constructed by covalently linking HTP and NAD^+ guided by catalytic mechanisms [9]. Using the Rosetta enzyme design application, we redesigned about 6 Å in TSA. Residue to evaluate binding energy. Command line parameters-field-detection-design-interface- cut1 0.0 -cut2 0.0 -cut3 10.0 -cut4 12.0 -cst_opt -chi_min -bb_min -cst_min -cst_design -design_min_cycles 2 -lig_packer_weight 1.8 -packing:use_input_sc -packing:soft_rep_design -packing:linmen_li 10 -nstruct 200 is applied and written in a “flag- file”. The Rosetta Enzyme Design application optimizes the catalytic position by combining the forces between the substrate and the important residues. The geometry of the transition state analog (TSA) was determined based on the catalytic mechanism and crystal structure. A Monte Carlo algorithm was used in the Rosetta Enzyme Design application to choose mutations and structural changes that minimize the overall energy and generate a redesigned 3D structure. The experimental data guided two rounds of study. In each round, the specific selected residues were subjected to saturation mutation, and the mutation with the most favorable binding energy was selected for experimental verification.

Reference

1. D.A. Case, K.B., I.Y. Ben-Shalom, S.R. Brozell, D.S. Cerutti, T.E. Cheatham, III, V.W.D. Cruzeiro, T.A. Darden, R.E. Duke, G. Giambasu, M.K. Gilson, H. Gohlke, A.W. Goetz,R Harris, S. Izadi, S.A. Izmailov, K. Kasavajhala, A. Kovalenko, R. Krasny, T. Kurtzman, T.S. Lee, S. LeGrand, P. Li, C. Lin, J. Liu, T. Luchko, R. Luo, V. Man, K.M. Merz, Y. Miao, O. Mikhailovskii, G. Monard, H. Nguyen, A. Onufriev, F. Pan, S. Pantano, R. Qi, D.R. Roe, A. Roitberg, C. Sagui, S. Schott-Verdugo, J. Shen, C.L. Simmerling, N.R. Skrynnikov, J. Smith, J. Swails, R.C. Walker, J. Wang, L. Wilson, R.M. Wolf, X. Wu, Y. Xiong, Y. Xue, D.M. York and P.A. Kollman. Amber 2020. University of California, San Francisco; 2020.
2. Case, D.A.; Aktulga, H.M.; Belfon, K.; Ben-Shalom, I.; Brozell, S.R.; Cerutti, D.S.; Cheatham III, T.E.; Cruzeiro, V.W.D.; Darden, T.A.; Duke, R.E. Amber 2021. University of California, San Francisco; 2021.
3. Jakalian, A.; Jack, D.B.; Bayly, C.I. Fast, efficient generation of high-quality atomic charges. AM1-BCC model: II. Parameterization and validation. *J Comput Chem* **2002**, *23*, 1623-1641. https://doi.org/10.1002/jcc.10128.
4. Jorgensen, W.; Chandrasekhar, J.; Madura, J.; Impey, R.; Klein, M. Comparison of Simple Potential Functions for Simulating Liquid Water. *J Chem Phys* **1983**, *79*, 926-935. https://doi.org/10.1063/1.445869.
5. Pearlman, D.A.; Case, D.A.; Caldwell, J.W.; Ross, W.S.; Cheatham, T.E.; DeBolt, S.; Ferguson, D.; Seibel, G.; Kollman, P. AMBER, a package of computer programs for applying molecular mechanics, normal mode analysis, molecular dynamics and free energy calculations to simulate the structural and energetic properties of molecules. *Computer Physics Communications* **1995**,

91, 1-41. https://doi.org/https://doi.org/10.1016/0010-4655(95)00041-D.

6. Andersen, H.C. Rattle: A "velocity" version of the shake algorithm for molecular dynamics calculations. *Journal of Computational Physics* **1983**, *52*, 24-34.

7. Chow, K.-H.; Ferguson, D.M. Isothermal-isobaric molecular dynamics simulations with Monte Carlo volume sampling. *Computer Physics Communications* **1995**, *91*, 283-289. https://doi.org/https://doi.org/10.1016/0010-4655(95)00059-O.

8. Le Grand, S.; Götz, A.W.; Walker, R.C. SPFP: Speed without compromise—A mixed precision model for GPU accelerated molecular dynamics simulations. *Computer Physics Communications* **2013**, *184*, 374-380. https://doi.org/https://doi.org/10.1016/j.cpc.2012.09.022.

9. Roe, D.R.; Cheatham, T.E., 3rd. PTRAJ and CPPTRAJ: Software for Processing and Analysis of Molecular Dynamics Trajectory Data. *J Chem Theory Comput* **2013**, *9*, 3084-3095. https://doi.org/10.1021/ct400341p.

10. Sanchez-Lengeling, B.; Aspuru-Guzik, A. Inverse molecular design using machine learning: Generative models for matter engineering. *Science* **2018**, *361*, 360-365. https://doi.org/10.1126/science.aat2663.
